# Laparoscopic pyeloplasty proficiency during a residency program after adoption of a standardized simulation training program is maintained during the COVID pandemic despite reduced surgery volume

**DOI:** 10.1590/S1677-5538.IBJU.2023.0021

**Published:** 2023-05-20

**Authors:** Antonio Rebello Horta Gorgen, Fernando Jahn da Silva Abreu, Artur de Oliveira Paludo, Mauricio Picolo Menegolla, Renan Timóteo de Oliveira, Patric Machado Tavares, Tiago Elias Rosito

**Affiliations:** 1 Hospital de Clínicas de Porto Alegre Serviço de Urologia de Urologia Porto Alegre RS Brasil Serviço de Urologia de Urologia, Hospital de Clínicas de Porto Alegre, Porto Alegre, RS, Brasil; 2 University of California Department of Urology Irvine CA USA Department of Urology, University of California Irvine, CA, USA;; 3 Universidade Federal do Rio Grande do Sul - UFRS Programa de Pós-Graduação em Ginecologia e Obstetrícia Porto Alegre RS Brasil Programa de Pós-Graduação em Ginecologia e Obstetrícia, Universidade Federal do Rio Grande do Sul - UFRS, Porto Alegre, RS, Brasil;; 4 Hospital de Clínicas de Porto Alegre Serviço de Cirurgia Geral Porto Alegre RS Brasil Serviço de Cirurgia Geral, Hospital de Clínicas de Porto Alegre, Porto Alegre, RS, Brasil;; 5 Universidade Federal do Rio Grande do Sul Faculdade de Medicina Porto Alegre RS Brasil Faculdade de Medicina, Universidade Federal do Rio Grande do Sul, Porto Alegre, RS, Brasil

**Keywords:** COVID-19, Surgical Procedures, Operative, Laparoscopy

## Abstract

**Purpose:**

To evaluate the effect of the standardized laparoscopic simulation training program in pyeloplasty, following its implementation and during the COVID-19 pandemic.

**Material and Methods:**

A retrospective chart review was performed at *Hospital de Clínicas de Porto Alegre*, a tertiary referral center in south Brazil, in which 151 patients underwent laparoscopic pyeloplasty performed by residents between 2006-2021. They were divided into three groups: before and after adoption of a standardized laparoscopic simulation training program and during the COVID-19 pandemic. The main outcome was a combined negative outcome of conversion to open surgery, major postoperative complications (Clavien-Dindo III or higher) or unsuccessful procedure, defined as need for redo pyeloplasty.

**Results:**

There was a significant reduction in the combined negative outcome (21.1% vs 6.3%), surgical time (mean 200.0 min vs 177.4 min) and length of stay (median 5 days vs 3 days) after the adoption of simulation training program. These results were maintained during the COVID-19 pandemic (combined negative outcome of 6.3%, mean surgical time of 160.1 min and median length of stay of 3 days) despite a reduction in 55.4% of the surgical volume.

**Conclusion:**

A structured laparoscopic simulation program can improve outcomes of laparoscopic pyeloplasty during the learning curve.

## INTRODUCTION

Ureteropelvic junction stenosis can be defined as a functional or anatomical obstruction of urine flow from the renal pelvis to the proximal ureter, which may result in renal symptoms or damage. Therefore, effective management is essential, requiring early identification of signs and symptoms, accurate diagnosis, and prompt treatment (1).

Correction of UPJ stenosis can be performed with three main treatments: open pyeloplasty, minimally invasive pyeloplasty, and endopyelotomy. Although open pyeloplasty is the traditional approach, minimally invasive pyeloplasty (including laparoscopic and robotic-assisted pyeloplasty) represents a less invasive option, providing relatively high success rates and low morbidity (1, 2).

Lately, laparoscopic pyeloplasty has become the gold standard for disease management in children and adults due to comparable efficacy with better aesthetic results, less postoperative pain, and less blood loss than open surgery (3). Also, laparoscopic pyeloplasty has significantly better results than endourological approaches (4). However, dismembered pyeloplasty is considered one of the most complex laparoscopic surgeries in urology and the learning curve associated with this procedure has not yet been investigated, and it is not clear whether proficiency in performing this surgical technique can be achieved during the training period. Some robotic pyeloplasty series suggest a learning curve of as many as 20 to 40 cases (5–7).

In the last years, several technological advancements were made in order to minimize errors and increase success rates. Robotic-assisted laparoscopy allows for better precision and may reduce the learning curve. Single-port surgery may minimize surgical trauma, has better patient satisfaction, faster recovery, and better aesthetic results, but fails to improve success, complication rates, or the learning curve. Improvements on laparoscopy such as 3D vision or articulated instruments may reduce surgical time and improve success, but there are no studies regarding a potential benefit on learning curve (8, 9).

To shorten the learning curve and reduce risks for the patient in the process, skills laboratories, virtual reality simulators, and box trainers can be used. Some studies have demonstrated that simulation training can improve basic laparoscopic skills and even reduce surgical time and complications in complex surgeries such as partial nephrectomies. (10)

During the COVID-19 pandemic, most hospitals drastically reduced elective procedures due to supersaturation of hospital beds and equipment. (11) Our hospital classified surgeries in groups (1 to 4) depending on the urgency and possibility of being postponed during the COVID-19 pandemic. Ureteropelvic strictures are not considered time-dependent surgeries and, in most patients, pyeloplasties can be postponed for a few months without significant damage. Therefore, our hospital classified pyeloplasty as group 4, which led to a significant reduction in pyeloplasty cases per year. However, simulation training was maintained during the COVID-19 pandemic (12).

Therefore, we hypothesized that the simulation training could improve the learning curve for laparoscopic pyeloplasty and even mitigate any possible drawbacks caused by the reduced surgical volume during COVID. For this reason, we compared the learning curve of pyeloplasties performed by residents before and after the adoption of a simulation training and during the COVID-19 pandemic. Our objective was to identify if the adoption of a structurized simulation training program might improve outcomes even with a reduced number of surgeries.

## MATERIAL AND METHODS

This study was approved by our Institutional Review Board under the number CAAE 18967819.8.0000.5327. We performed a retrospective review of patients submitted to laparoscopic pyeloplasty at our hospital from 2006 to 2021 performed by last year residents (PGY-5) under the supervision of a senior urologist. Since 2005 we have routinely performed laparoscopic pyeloplasty using the Anderson-Hynes technique for all patients with UPJ stenosis (except infants with less than 1 year old). All patients submitted to laparoscopic pyeloplasty performed by a PGY-5 resident were included. Exclusion criteria included patients with insufficient data, patients with kidney malformations (i.e. pelvic kidney) that could affect surgical outcomes, and patients with a follow up shorter than 6 months.

For the analysis of the standardized laparoscopic simulation training program, pyeloplasties were classified into three groups:

- Group 1 (before simulation training program) included procedures from 2006 to February 2017;
- Group 2 (after simulation training program) included procedures from March 2017 to February 2020;
- Group 3 (during COVID-19 pandemic) included patients from March 2020 to February 2022.

This was because the first PGY-5 residents who completed the simulation training program started their final urology year in March 2017 (starting month of residency in Brazil). The simulation training program, performed at *Instituto Simutec*, consisted of up to 244 hours of training in box trainers and in virtual simulation, which included basic skills (instruments manipulation), laparoscopic activities (peg transfer, cutting, electrocautery, etc.), suturing and performing virtual nephrectomies. The whole program was previously described by our group in another study (10). The COVID-19 pandemic surgical protocol was implemented in the second half of March 2020 and ended in April 2022.

The main endpoint was a combined negative outcome of conversion to open surgery, major postoperative complications (Clavien-Dindo III or higher) or unsuccessful procedure defined as the need of redo pyeloplasty. Secondary outcomes were surgical time and length of stay.

Analyses were performed using Excel^®^ 2013 and IBM SPSS Statistics v.25 software. The significance level adopted was 0.05. Categorical variables were evaluated by absolute and relative frequency. A chi-square test or exact Fisher test was used to compare proportions for the studied variables among the three periods. For the quantitative variables, the Shapiro-Wilk normality test was initially performed. Those variables with a symmetrical distribution are presented as the mean and standard deviation, while the other quantitative variables are presented as the median and interquartile range (median (p25, p75)). To compare the mean values of each variable between the three periods, analysis of variance (ANOVA) was performed.

## RESULTS

During the study period, there were 174 laparoscopic pyeloplasty, of which 23 were excluded (follow-up on a different institution or surgery performed by someone other than a PGY-5 resident). We included 151 laparoscopic pyeloplasties performed by PGY-5 residents in this study.

Of these 151 procedures, 90 were before the adoption of the simulator program (group 1), 47 were after (group 2) and 14 were during the COVID-19 pandemic (group 3). Mean age at the time of surgery was 31.0 years and 57.0% of patients were male. There was no significant difference between the groups. The main reason for surgery was pain in all three groups. There was a higher yearly surgical volume for group 2 (mean of 15.7 surgeries/year) compared to groups 1 (8.2) and 3 (7.0). During all years, three residents exclusively performed those procedures, with a mean of 2.7, 5.2 and 2.3 cases per resident in each group respectively. Table-1 presents complete data for the three groups.

**Table 1:** Characteristics of patients submitted to laparoscopic pyeloplasty before and after adoption of the simulation training program and during COVID-19 pandemic.

		Group 1	Group 2	Group 3	p
		Before Simulation	After Simulation	During COVID	
Cases per year - mean		8.2	15.7	7.0	*
Age - mean (SD)		32.3 (16.4)	29.8 (19.7)	26.9 (20.4)	0.4946
Sex (male) - % (n)		56.7% (51/90)	61.7% (29/47)	42.9% (06/14)	0.4561
Side (right) - % (n)		53.3% (48/90)	44.7% (21/47)	42.9% (06/14)	0.5458
**Surgical indication - % (n)**					
	Pain	76.7% (69/90)	61.7% (29/47)	85.7% (12/14)	0.7176
	UTI	4.4% (04/90)	10.6% (05/47)	7.1% (01/14)	
	Lithiasis	5.6% (05/90)	4.3% (02/47)	7.1% (01/14)	
	Multiple symptoms	6.7% (06/90)	12.8% (06/47)	7.1% (01/14)	
	Severe Hydronephrosis	7.8% (07/90)	10.6% (06/47)	7.1% (01/14)	
Creatinine - mean (SD)		0.89 (0.31)	0.99 (0.40)	0.94 (0.35)	0.3572
DMSA - mean (SD)		18.7 (8.6)	16.9 (7.6)	12.5 (7.3)	0.1468
Relative DMSA - mean (SD)		40.0 (13.4)	40.7 (14.1)	36.1 (19.6)	0.7396
Previous UPJ surgery* - % (n)		7.8% (08/90)	17.0% (08/47)	7.1% (01/14)	0.3158

* Not enough data to perform an ANOVA test

SD = Standard deviation; UTI = Urinary tract infection; DMSA = Dimercapto succinic acid renal scan

There was a statistically significant reduction in the combined negative outcome after adoption of the structured program. In comparing group 1 vs. group 2 and 3 together (i.e. residents who didn’t have simulation training vs. residents who did), the CNO reduced from 21.1% to 6.2% (p = 0.009). When analyzing as three separate groups, statistical significance improvement remained, reducing the combined negative outcome from 21.1% (19 patients) on group 1 to 6.4% (3 patients) on group 2 and 7.1% (1 patient) on group 3 (p = 0.0443). The rate of the combined negative outcome remained the same during COVID-19 pandemic compared to group 2. Surgical time was also improved on group 2 (mean = 177.4min) and group 3 (mean = 160.1min) compared to group 1 (mean = 200.0min) (p = 0.0127). Length of stay was shorter on group 2 (median = 3 days) and group 3 (median = 3 days) compared to group 1 (median = 5 days) (p = 0.0040). Table-2 shows complete data regarding these outcomes for each group.

**Table 2:** Surgical outcomes before and after adoption of the simulation training program and during the COVID pandemic.

	Group 1	Group 2	Group 3	p
	Before Simulation	After Simulation	During COVID	
CNO	21.1% (19/90)	6.4% (03/47)	7.1% (01/14)	**0.0443**
Conversion	7.8% (07/90)	4.3% (02/47)	0%	0.6656
Major complication	8.9% (08/90)	0%	0%	0.0769
Redo Pyeloplasty	4.4% (04/90)	2.1% (01/47)	7.1% (01/14)	0.4288
Surgical Time - mean	200.0min (SD 58.2)	177.4min (SD 59.1)	160.1min (SD 49.6)	**0.0127**
LOS - median	5.0	3.0	3.0	**0.0040**

CNO = Combined negative outcome (Conversion, Major Complication or Redo Pyeloplasty); LOS = Length of Stay; SD = Standard deviation

The median follow-up was 18 months (6 - 179 months). Follow-up was longer for group 1 (median 30 vs 10 vs 8.5 months), but in all groups the minimum follow-up was 6 months.

## DISCUSSION

The use of laparoscopic standardized simulation training has been associated with improvement in surgical outcomes (10, 13). However, no studies were found on simulation training for pyeloplasties. Also, comparing two different time-periods may have other significant factors that could influence the surgical outcomes. Therefore, our goal with this study was to identify if the simulation program improved outcomes and if these results were maintained during a reduced surgical volume period (COVID-19 pandemic) (12).

Our study demonstrated a statistically significant improvement for all analyzed outcomes during the residency learning curve with the introduction of the simulation training: combined negative outcomes, surgical time, and length of stay. However, other factors may have influenced these results, such as higher experience of the attending urologists and higher laparoscopic surgical volume of the residents. Relles et al. studied the effect of resident surgical volume on pancreaticoduodenectomy and found that as residents perform more cases, there were fewer complications (14). On the other hand, Sellers et al. demonstrated that university-based residencies had higher surgical volume than non-university-based residencies but there was no difference in mortality or complications after matching the cohorts (15). Elkbuli et al. showed that operative confidence increased during residency when residents had a higher surgical case volume (16).

Several studies have shown concern about the impact of COVID-19 on medical education (17–20). Purdy et al. published a study that showed a significant reduction in surgical volume within the first 4 months of the COVID-19 pandemic, which could be a concern due to its effect on surgical training (17). Our group has also previously published a study on the first three months of the COVID-19 pandemic, showing a reduction of 63.4% of all surgeries, especially in non-oncology areas (12). Prezotti et al. reported a survey with Brazilians residents showing that most senior residents would prefer to extend their residency to compensate for reduced surgical training (21) Our third group was very small (only 14 pyeloplasty were performed during the two years period). If we grouped it together with group two (i.e. all residents who did trained vs. all residents who didn’t do simulation training), our results would still be significant, as group 2 and 3 were very similar. However, by separating in groups 2 and 3, we demonstrated that, aside from COVID-19 severely impacting surgical volumes, surgical outcomes were maintained compared to previous years and were better than before the implementation of the simulation training program. During COVID-19, residents had more free time and could train more on simulation. We believe this had a positive impact and managed to keep the adequate formation of surgical residents during the pandemic.

Regarding pyeloplasty proficiency, a few studies have already been performed. However, this study is among the largest series reported to date, and to our knowledge, the largest that only analyzes procedures performed entirely by residents.

Singh et al. in 2010 evaluated their first 100 cases of laparoscopic pyeloplasty and demonstrated a reduction in surgical time and complications after the first 50 cases (22). Tasian et al. in 2013 published a study analyzing the results of 4 fellows during their learning curve. They demonstrated an average 3.7-minute decrease in surgical time per case and estimated a learning curve of 37 cases. However, their study participants were fellows and therefore probably had some experience before (23). Our study had 46 different residents with an average of 3.3 (1–10) pyeloplasty cases per resident. Due to the small average number of laparoscopic pyeloplasties, it was not possible to evaluate individual learning curves, but as a group, such a small number of cases per year was enough to reach surgical time, complications, and success rates similar to that found in the literature.

Szydelko in 2011 evaluated 150 laparoscopic pyeloplasty complication rates based on the Clavien-Dindo classification during the learning curve of two surgeons. Success rate was 90.5%, intraoperative complications occurred in 6%, and major complications (Clavien-Dindo ≥3) in 9.2% (24). Arap et al. in 2013 evaluated 90 pyeloplasties performed by residents and analyzed their outcomes. Mean operative time was 222 min, conversion rate was 3.4%, major complications rate was 4.4%, and mean length of stay was 3.49 days (25). In our study, we have shown similar rates in the group before simulation, and after simulation the residents had better results even during their small learning curve.

In our opinion, our study not only demonstrated better results after the implementation of the simulation program, but also demonstrated that similar results can be achieved even in lower surgical volume periods. The low mean number of cases per resident was the main limitation of our study, as it did not allow us to individually assess the learning curve. Another limitation is the retrospective aspect and the possibility of other factors that could have influenced these results, such as attending experience, prior laparoscopic experience, and better management of patients. However, this limitation has been mitigated because it would be expected to be different in the group during COVID-19 pandemic.

## CONCLUSION

The implementation of a structured laparoscopic simulation program can improve the outcomes of laparoscopic pyeloplasty during the learning curve, reducing surgical time, negative outcomes (conversion to open surgery, complication rate or failure needing a secondary pyeloplasty) and length of stay. This improvement was maintained even during the reduced surgical volume during the COVID-19 pandemic.
